# Complementary and alternative medicine use among cancer patients in Iran: A systematic review

**DOI:** 10.1016/j.pmedr.2024.102644

**Published:** 2024-02-08

**Authors:** Mohammad Yousefi, Hamid Reihani, Mojtaba Heydari, Ramin Nasimi Doost Azgomi, Mohammad Hashem Hashempur

**Affiliations:** a Student Research Committee, School of Medicine, Shiraz University of Medical Sciences, Shiraz, Iran; b Poostchi Ophthalmology Research Center, Department of Ophthalmology, School of Medicine, Shiraz University of Medical Sciences, Shiraz, Iran; c Traditional Medicine and Hydrotherapy Research Center, Ardabil University of Medical Sciences, Ardabil, Iran; d Research Center for Traditional Medicine and History of Medicine, Department of Persian Medicine, School of Medicine, Shiraz University of Medical Sciences, Shiraz, Iran

**Keywords:** Complementary therapies, Traditional Persian medicine, Public health, Cancer, Neoplasm, Iran, Systematic review

## Abstract

**Background:**

Cancer patients frequently turn to complementary and alternative medicine (CAM) to mitigate the adverse effects of conventional cancer treatments, alleviate stress, manage comorbidities, and address the cancer itself. This systematic review aims to investigate descriptive studies conducted in Iran, focusing on the prevalence and methods of CAM use among cancer patients.

**Methods:**

A thorough search of PubMed, Scopus, Google Scholar, Web of Science, Magiran, and the Scientific Information Database (SID) was conducted in both English and Persian from inception to July 2023. Inclusion criteria targeted studies exploring the frequency and patterns of CAM utilization among Iranian patients across diverse cancer types.

**Results:**

A total of 604 articles were found in the preliminary search, and 14 were included in this review. Among the 3,990 participants in the included studies, a weighted average of 51.83% (range 4.6%-100%) had used at least one CAM method. The most commonly used CAM methods were prayer (41.8%), medicinal herbs (30.1%), traditional and folk treatments (27.2%), bloodletting (17.3%), and hydrotherapy (13%). Patients reported using CAM for various reasons, including their beliefs, desire to cure the disease, reduce stress, positive past experiences, and improve physical condition. On average, 39% of patients informed their physicians about their CAM use.

**Conclusion:**

The findings underscore the relatively prevalent adoption of CAM by Iranian cancer patients. Given the low rate of disclosure to physicians, there is a compelling need for healthcare system vigilance and monitoring to ensure comprehensive patient care.

## Highlights

• 51.83% of the participants reported using complementary and alternative medicine (CAM), with usage ranging from 6.4% to 100%.
• The frequency of CAM techniques used by cancer patients was as follows: prayer 41.8%, medicinal herbs 30.1%, traditional and folk treatment 27.2%, bloodletting 17.3%, hydrotherapy 13%, massage 7.6%, cupping therapy 6.8%, energy therapy 5.1%, yoga 5.1%, leech therapy 4.5%, homeopathy 2.6%, acupuncture 1.1%, acupressure 0.6%, hypnotism 0.4%.
• The primary reasons for using CAM among cancer patients included their beliefs, seeking a cure for the disease, stress reduction, positive past experiences, improvement of physical condition, safety of the methods, suggestions from family and friends, and reduction of side effects from conventional treatments such as fatigue, nausea and vomiting, and diarrhea.
• Among the studies, the disclosure to physicians was reported at 39%.

## Graphical abstract

**Figure:**
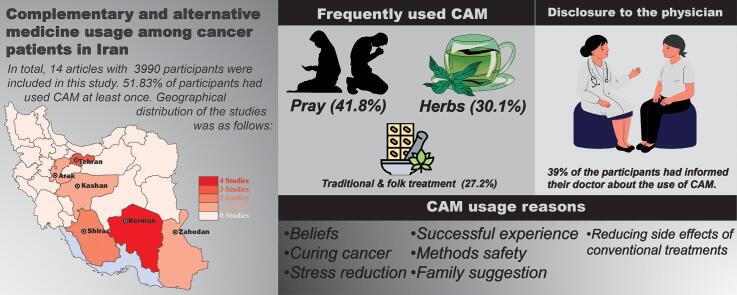

## 1 Introduction

Complementary and alternative medicine (CAM) refers to a set of knowledge and skills based on experiences and native beliefs of different cultures, which is used to prevent, diagnose, and treat physical and mental illnesses, as defined by the World Health Organization (WHO) (World Health Organization, 2019). CAM methods encompass a wide range of options, including medicinal plants, energy-based therapies, homeopathy, and body-based practices, and refer to any health intervention not included in conventional medicine (Clarke et al., 2015). The nativeness, holistic approach, and cost-effectiveness of CAM methods have contributed to their widespread use in recent years (Hashempur et al., 2018). The popularity of CAM methods varies by culture and geographic region, with some methods being more common in certain areas than others (Zörgő et al., 2018). Consequently, various traditional medicine schools, such as Indian, African, Chinese, and Persian medicine, have emerged (Shakeri et al., 2020). In this study, we focus specifically on studies conducted in Iran to consider these influential factors.

Previous investigations have shown that adults with chronic conditions are more likely to use CAM compared to those who do not suffer from chronic illnesses (Berna et al., 2019). Cancer, which is responsible for 15 % of annual mortality in Iran and ranks third after accidents and cardiovascular problems, is one of the main chronic conditions that lead patients to use CAM (Wode et al., 2019, Bari et al., 2021). Moreover, the high side effects of conventional cancer treatments, such as chemotherapy, surgery, and radiotherapy, also encourage patients to use CAM (Keene et al., 2019, Jones et al., 2019, Finnegan-John et al., 2013 Jul). The high prevalence, chronicity, and complications of cancer treatment increase the probability of CAM use and highlight the importance of research in this population.

This study aims to review the descriptive studies conducted in Iran on the frequency and methods of CAM use by cancer patients. To the best of our knowledge, this study is the first systematic review on this topic in Iran. Given that previous review studies included only a small number of Iranian studies (Horneber et al., 2012), this study aims to address potential gaps in the literature.

## 2 Methods and materials

### 2.1 Search strategy

We conducted a comprehensive search of the literature to identify appropriate keywords for the study. Using these keywords, we designed a search strategy for each of the following databases: PubMed/Medline, Scopus, Google Scholar (in English and Persian), Web of Science, Magiran (in Persian), and Scientific Information Database (SID) (in Persian). Our final search was performed in July 2023. An example of the search syntax used in the PubMed database is presented below: ((“Complementary Therapies”[MeSH Terms] OR “Medicine, Traditional”[MeSH Terms] OR (“Complementary medicine”[Title/Abstract] OR “Alternative medicine”[Title/Abstract] OR “Alternative therapy”[Title/Abstract] OR “Traditional medicine”[Title/Abstract])) AND (“Neoplasms”[MeSH Terms] OR (“Cancer”[Title/Abstract] OR “Neoplasm*”[Title/Abstract])) AND (“Iran”[MeSH Terms] OR “Iran”[Title/Abstract])) NOT “Clinical Trial”[Publication Type].

### 2.2 Eligibility criteria

The eligibility criteria for this study were: (a) descriptive studies, (b) studies conducted in Iran, and (c) studies that examined the prevalence and pattern of CAM use in cancer patients. Clinical trials, review studies, and studies conducted outside Iran or reported in languages other than Persian or English were excluded from the review.

### 2.3 Literature screening and evaluation process

The protocol of this study has been reviewed and approved by the Medical Ethics Committee of Shiraz University of Medical Sciences by approval ID: IR.SUMS.MED.REC.1401.307. The literature screening and evaluation process was conducted meticulously (by M.Y. and R.N.D.A.) according to the methods previously explained (de Almeida and Schlechta Portella, 2018). A systematic approach was employed, involving the comprehensive search of databases. A total of 604 articles were identified in the preliminary search, and the subsequent screening process involved a thorough examination of titles, abstracts, and full texts to identify studies meeting the inclusion criteria.

### 2.4 Selection process

The results of our search were extracted in the appropriate format for EndNote X7 reference management software and transferred to it. Duplicate studies were then removed. Two researchers (M.Y. and R.N.D.A.) independently executed the title and abstract screening process, and the selected studies were screened again by full-text review. Any disagreement during the review process was resolved by the study supervisor (M.H.H.). Additionally, the reference sections of included articles were reviewed to identify potentially absent studies in the systematic search.

### 2.5 Data collection

Two researchers (M.Y. and H.R.) reviewed the selected studies in detail, and the following variables were extracted: first author, publication year, study type, study location (city, country, research center), study aim, sample size, response rate (number and percentage), inclusion and exclusion criteria, questionnaire description, participant demographic information, diagnosis date, cancer type, frequency of using different CAM techniques, frequency of the three most common types of CAM used, CAM utilization duration, source of knowledge about CAM, the physician's awareness of CAM usage by the patient, and significant relationships between the characteristics of the studied population and CAM use.

### 2.6 Quality assessment

We used the quality assessment tool (QAT) designed by Bishop et al. (2010) to evaluate the quality of included articles. It should be noted that this tool has been used in other review articles, in addition to the study by (Grant et al., 2012, Italia et al., 2014). According to the tool's criteria, each article can receive a maximum of 17 scores. However, since the topic of using CAM in cancer patients is generally retrospective and all reviewed articles in this study are also retrospective, we modified the maximum score of this tool to 16.

## 3 Results

### 3.1 Study selection and characteristics

**Fig. 1:**
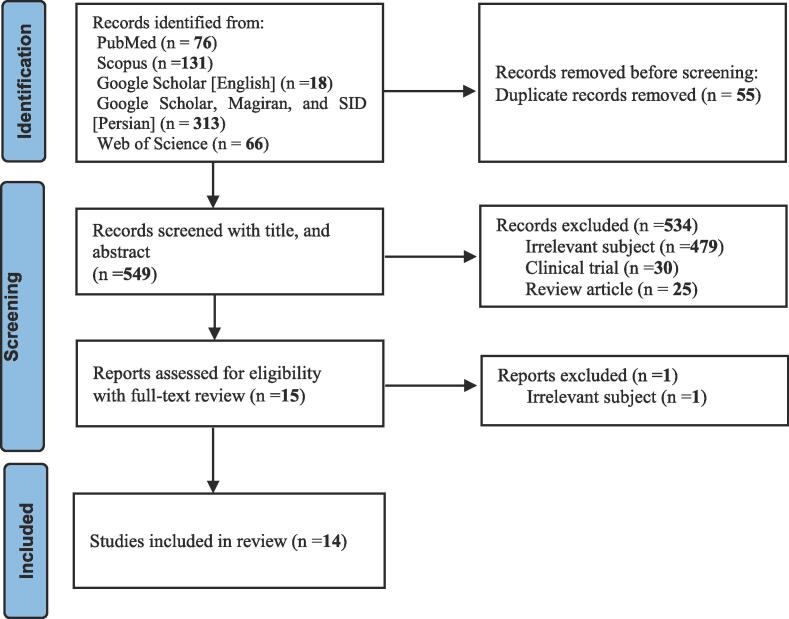
Preferred Reporting Items for Systematic Reviews and Meta-Analyses (PRISMA) flow chart demonstrating the selection process of enrolled articles in the systematic review.

Out of 604 articles entered by our search strategy, 14 studies were finally selected. The selection process is demonstrated in Fig. 1. Characteristics of the included studies are presented in Table 1. These results indicate that since 2005, the subject of using CAM by cancer patients has been addressed in Iran. Kerman city had the largest number of studies. The response rate of the studies varied from 36 % to 100 %. Except for the study of Bordbar et al. (2016), the rest of the studies were conducted on adults. Breast cancer was the most frequent cancer among the samples. In the following, the specific information related to the use of CAM in each of the studies is collected in Table 2. A significant portion of cancer patients use CAM. Prayer has been one of the major methods used in Iran. The positive experience of patients and their relatives had a significant impact on CAM usage. Although the use of CAM among cancer patients is high, few disclose it to their doctors. Researchers have used three methods in these studies to obtain information (questionnaire, interview, and combination of both).

**Table 1:** Aims, location, and cohorts’ characteristics of the included studies in the systematic review.

**Author, year**	**Study aims**	**Location**	**Sample size (M/F)** **(Response rate)**	**Participants' mean age (SD)**	**Cancer type (%)**
(Montazeri et al., 2005)	Investigating the relationship between anxiety, depression and the quality of life of cancer patients using CAM	NR	177 (0/177) (100 %)	47.73 (NR)	Breast (1 0 0)
Sajadian et al., 2005)	Examining the common methods and determining the frequency and reasons for cancer patients to refer to such CAM treatment methods	Tehran, Imam Khomeini Hospital, and Novin Medical Radiation Institute (NMRI)	625 (203/422) (100 %)	47.61 (15.1)	Breast (28), gastrointestinal (25), and urogenital (14), head and neck (9), hematological (7), other (17)
(Montazeri et al., 2007)	Investigating factors affecting the use of CAM among cancer patients	Tehran, Tehran cancer Institute	625 (203/422) (100 %)	47.6 (NR)	Gastrointestinal (27), breast (26), and urogenital (12), head and neck (10), other (16)
(Sajadian et al., 2009)	The rate of use of different fields of CAM except prayer therapy in cancer patients	Tehran, Imam Khomeini Hospital, and Novin Medical Radiation Institute (NMRI)	625 (203/422) (100 %)	47.61 (15.1)	Breast (27.5), gastrointestinal (25), and urogenital (14)
(Bordbar et al., 2016)	Frequency and characteristics of CAM use in pediatric oncology patients	Shiraz, two separate pediatric referral centers	150 (86/49) (90 %)	7.8 (4.7)	NR
(Amirmoezi et al., 2017)	Determining the use of CAM and its related factors in Iranian cancer patients	Shiraz, Amir oncology center	98 (17/19) (36.7 %)	39.3 (13.2)	Hematologic malignancies, solid tumors
(Bazrafshani et al., 2019)	Determining the amount and reasons for using medicinal plants in cancer patients	Kerman, two cancer clinics	315 (94/221) (100 %)	51.46 (14.01)	Breast (37.7), gastrointestinal (19), lymphoma and hematologic tumors (17), gynecologic (10), thorax (5), other (7), unclear (1.9)
(Dehghan et al., 2019)	Frequency of CAM use in cancer patients in Kerman	Kerman, cancer clinic, and Yas Association of Kerman	300 (88/93) (60 %)	49.64 (16.91)	Leukemia (23.2), gastrointestinal (14.9), bone marrow (14.9), genital (13.8), other (33.2)
(Farahani et al., 2019)	The opinion of cancer patients about the use of CAM	Thirteen cities in Iran (not exactly specified)	176 (96/80) (100 %)	53.14 (13.65)	NR
(Jalili et al., 2020)	Measuring the use of chemical and herbal medicines for symptomatic treatment in patients receiving chemotherapy	Kashan, Shahid Beheshti and Yasrebi Hospitals	186 (91/95) (100 %)	58.1 % aged more than 50	Breast (23.1), colon (21), bones (11.3), lung (7), prostate (3.8), cervix (3.8), liver (3.2), lymph nodes (2.7), brain (8.06), kidney (5.37), testis (4.3), and Hodgkin lymphoma (6.45)
(Nejat et al., 2022)	Frequency of CAM use in cancer patients in Arak	Arak, Ayatollah Khansari Center	320 (161/159) (100 %)	55.11 (15.8)	Leukemia (25.9), breast (13.4), intestine (11.5), lung (9.7), stomach (6.9), bone (6.9), liver (5.6), prostate (3.1), brain (2.8), kidney (2.2), esophagus (1.6), pancreas (0.3), other (10.3)
(Dehghan et al., 2022)	Investigating the relationship between the use of CAM and the quality of life of cancer patients	Kerman, Bahonar hospital, Javad Al-A’meh center, and doctors’ offices	300 (107/131) (79 %)	51.07 (14.01)	The majority of female patients had breast or ovarian cancer, whereas the majority of male patients had blood cancer
(Dehghan et al., 2022)	Investigating the relationship between the use of CAM and psychosomatic symptoms in terminally ill cancer patients	Kerman, two centers, and doctors’ offices	250 (100/121) (88.4 %)	51.66 (13.34)	Breast (33.9), blood (26.2), gastrointestinal (11.8), lung (10.9), ovary (10.4), other (6.8)
(Asadi Sarvestani et al., 2022)	Investigating the use of CAM and informing the doctor in cancer patients	Zahedan, Imam Ali Hospital, Khatam Hospital, and an oncologist’s office	146 (0/130) (89 %)	36.11 (8.75)	Breast (29.2), stomach (26.9), cervix (23.1), blood (10), skin (6.2), other (4.5)

SD: standard deviation; NR: not reported; CAM: complementary and alternative medicine.

**Table 2:** Main reports related to complementary and alternative medicine use in the included studies of the systematic review.

**Author, year**	**Prevalence of CAM usage (%)**	**Three most frequently used CAM (%)**	**CAM use reasons (%)**	**Disclosing CAM use to their physician**	**Variables that increase the probability of CAM use**
(Montazeri et al., 2005)	32.2	Prayer and spiritual healing (73.8), bio-energy (11.5), homeopathy (3.3), herbs (3.3)	NR	NR	Severe depression (p = 0.04, CI 95 % 1.06–5.89, and OR = 2.49), time since diagnosis (p = 0.007
(Sajadian et al., 2005)	35	Prayer and healing (81), energy therapy (9), homeopathy (7)	Beliefs (70), receiving hope (17), improving physical condition, general condition and increasing energy (6), methods safety (6)	50	Married (p < 0.0001), older age (p < 0.02), lower education level (P < 0.0001)
(Montazeri et al., 2007)	35.04	Prayer and spiritual healing (75.7), bio-energy (8.5), homeopathy (6.4)	NR	NR	Fear of cancer recurrence (OR = 2.03, 95 % CI = 1.45–2.85, p < 0.0001), dissatisfaction with their care physician (OR = 1.98, 95 % CI = 1.36–2.89, p < 0.0001)
(Sajadian et al., 2009)	6.4 (except prayer)	Energy therapy (20), homeopathy (15), herbal therapy (7)	methods safety (21), improvement of physical condition and general mood (16), receiving hope (13), good relationship with the therapist and answering vague complaints (10)	50	Age below 40 (p = 0.05), higher education (p = 0.0001), employee occupation (p = 0.006)
(Bordbar et al., 2016)	84.4	Faith healing (92.6), Zinc (43.7), Leek (37)	Increased immunity, improvement and cure of patient, increased appetite and growth, sense of well-being	49.1	Nothing significant
(Amirmoezi et al., 2017)	94.4	Prayer (86.1), medicinal plants (41.7) and nutritional supplements (multivitamin and vitamin C) (33.3)	Physician recommendation, improvement of general health and emotional well-being, relieving pain especially bone pain, anemia, feeling relaxed, improvement of gastrointestinal discomfort	NR	NR
(Bazrafshani et al., 2019)	84.1	Matricaria chamomilla L. (71.9), Mentha aquatica (71.9), Malva sylvestris L. (70.8)	Successful experience in previous uses (64), curing the disease and improving the quality of life (40.2), suggesting by family and friends (39), reducing the side effect of medications (26.9), dissatisfaction with physician and chemotherapy treatment (3)	16.1	High-school diploma or more (OR: 2.01; 95 % CI, 1.02–3.97; p = 0.04), residence in urban areas (OR, 2.56; 95 % CI, 1.30–5.05; p < 0.0001), unclear metastatic status (OR, 0.19; 95 % CI, 0.05–0.71; p = 0.01), and constipation and diarrhea (OR, 2.11; 95 % CI, 1.09–4.05; p = 0.02), skin lesions (OR, 2.09; 95 % CI, 1.10–3.98; p = 0.02)
(Dehghan et al., 2019)	96.1 (including prayer and Vow), 45.9 (except prayer and Vow)	prayer (92.6), Vow (85.1), herbal medicine (44.2)	herbal medicine/prayer/Vow: treatment (62.5/89.2/75.3), reducing complications of treatment (27.5/16.2/12.3), reducing stress and anxiety (12.5/8.4/8.4), other reasons (46.3/25.7/42.2)	37.50 %	Living in the city (p = 0.03), female gender (OR = 2.11, CI = 1.01–4.04, p = 0.025)
(Farahani et al., 2019)	65.6	Medicinal herbs (48.3), nutritional counseling (39.8), traditional & folk treatment (27.3), touch and movement therapies (yoga) (27.3)	Increase the general ability to resist the disease, improve daily performance, and reduce the complications of chemotherapy, decrease the symptoms of anxiety, fatigue, depression, spiritual distress, pain, problems with daily tasks, dyspnea, weight change, and sleep problems	51.71	NR
(Jalili et al., 2020)	87.4	Zingiber officinale (38.7), Mentha (36), Cichorium intybus (30)	Fatigue (89.2), dry mouth (63.9), insomnia (62.4), anorexia (61.8), pain (61.3), Constipation (54.8), low mood (52.7), nausea (46.8), dysuria (35.5), mouth ulcer (32.8), dyspnea (32.3), cough (28), diarrhea (27.4), vomiting (24.2)	NR	NR
(Nejat et al., 2022)	44.3	visiting holy places (22.5), yoga (20), prayer (14)	Improve physical conditions (73.2), reduce pain (25), anxiety (11.3), depression (7.3), constipation (7.3), insomnia (4.8), anorexia (3.2)	NR	NR
(Dehghan et al., 2022)	85.7	Prayer (81.5), medicinal herbs (33.6), massage (9.2)	Reducing physical complications of the cancer and its treatment, reducing stress and anxiety	nutritional supplements (90), wet cupping (66.67), dry cupping (60), and medicinal plants (5)	Social aspect of QOL (p = 0.02), the global quality of life score (p = 0.003), no sleep disturbance (p = 0.04), nausea and vomiting (p = 0.02), not having dyspnea (p = 0.01), constipation (p = 0.03), no financial impact (p = 0.02)
(Dehghan et al., 2022)	87.3	Prayer (83.7), medicinal herbs (35.8), massage (9.5)	Reducing physical complications of the cancer and its treatment, reducing stress and anxiety	dietary supplements (62.5), wet cupping (42.9), dry cupping (20), medicinal plants (0)	Higher activity (p = 0.02), nausea (p = 0.03), drowsiness (p = 0.01), well-being (p = 0.02), not having shortness of breath (p = 0.03)
(Asadi Sarvestani et al., 2022)	100 (CAM usage was inclusion criteria)	TPM: Herbal Medicines (98.5), medicinal plants (94.6), Herbal Distillates (70.8). CAM: massage (16.2), hydrotherapy (13.1), yoga (8.5)	NR	19.23	Higher monthly income (p = 0.009, Pearson correlation = 0.228)

NR: not reported; CAM: complementary and alternative medicine; TPM: traditional Persian medicine; QOL: quality of life.

Regarding the used questionnaires, seven studies used questionnaires from previous studies, three were designed by study researchers, and four studies did not report detailed information in this area. In terms of geographic distribution, Kerman, Tehran, and Shiraz each had 4, 3, and 2 studies, respectively. Other cities, including Arak, Kashan, and Zahedan, had one study each. In addition, one multi-center study was conducted in collaboration with universities of 13 different cities (Farahani et al., 2019), and one study did not mention the center(s) for data gathering (Montazeri et al., 2005).

### 3.2 Patients’ demographics

A total of 4293 patients were questioned in the enrolled studies, of which 3990 answered. Therefore, the overall response rate was 92.94 %. Out of 14 articles, only one study did not specify the average age of the participants (Jalili et al., 2020). Also, only one study was conducted in the pediatric age group, which reported an average age of 7.8 ± 4.7 years old (Bordbar et al., 2016). The rest of the studies were conducted on adults, whose mean age was between 36.11 and 55.11 years (total mean of 48.47 years old). Regarding gender distribution in the respondents, two studies were only conducted on female patients (Montazeri et al., 2005, Asadi Sarvestani et al., 2022). Overall, 1449 (36.3 %) patients were male, and 2541 (63.7 %) were female. 22.52 % of the patients had breast cancer, 16.7 % had gastrointestinal cancer, and 6.5 % had urogenital cancer.

### 3.3 Complementary and alternative medicine

#### 3.3.1 Prevalence, satisfaction, and duration of the CAM usage

In total, 51.83 % of the participants have used CAM, which ranged from 6.4 % to 100 %. Among the 14 studies, only 4 reported the level of satisfaction, which varied from 13 to 100 %.

Another criterion that was different in different studies was the duration of CAM use. Two articles did not specify it at all (Bordbar et al., 2016, Jalili et al., 2020). On the other hand, Nejat et al.'s study reported an average of 11.47 ± 4.02 months (Nejat et al., 2022). Two studies considered the time of CAM use since diagnosis or during treatment (Asadi Sarvestani et al., 2022, Bazrafshani et al., 2019).In addition, four studies were considered this time during one year (Farahani et al., 2019, Dehghan et al., 2019, Dehghan et al., 2022, Dehghan et al., 2022), and two studies during six months leading to the completion of the questionnaires (Sajadian et al., 2005, Sajadian et al., 2009). Finally, three of the articles considered the use of CAM lifelong (Montazeri et al., 2005, Montazeri et al., 2007, Amirmoezi et al., 2017).

#### 3.3.2 CAM methods, usage reasons, and disclosing it to the physician

**Fig. 2:**
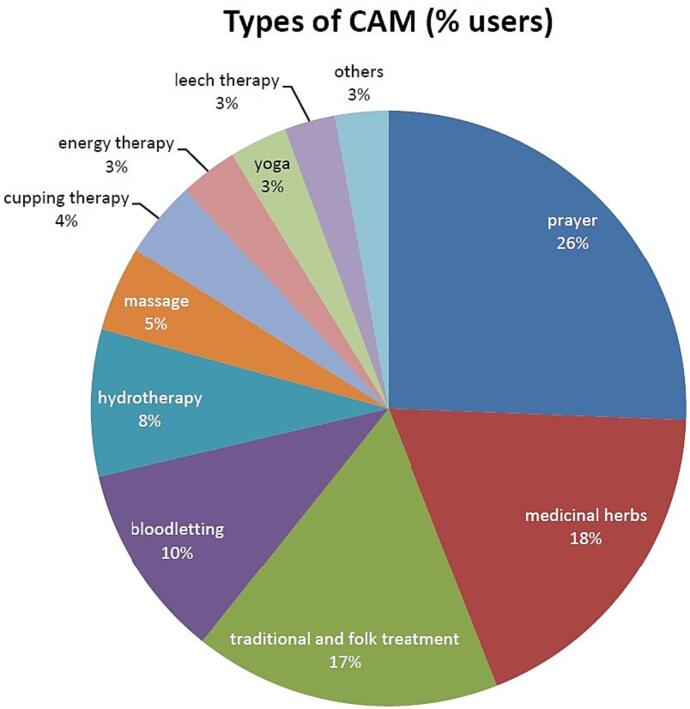
Prevalence of different types of complementary and alternative medicine (CAM) among cancer patients who classified as CAM users.

The frequency of using CAM techniques by cancer patients was as follows: prayer 41.8 % (1331 patients/3183 respondents), medicinal herbs 30.1 % (1013 patients/3365 respondents), traditional and folk treatment 27.2 % (48 patients/176 respondents), bloodletting 17.3 % (78 patients/450 respondents), hydrotherapy 13 % (17 patients/130 respondents), massage 7.6 % (96 patients/1261 respondents), cupping therapy 6.8 % (75 patients/1090 respondents), energy therapy 5.1 % (131 patients/2529 respondents), yoga 5.1 % (144 patients/2813 respondents), leech therapy 4.5 % (29 patients/631 respondents), homeopathy 2.6 % (73 patients/2714 respondents), acupuncture 1.1 % (24 patients/2028 respondents), acupressure 0.6 % (5 patients/722 respondents), hypnotism 0.4 % (6 patients/1257 respondents) (Fig. 2).

In addition, the main reasons for using CAM in cancer patients were their beliefs, curing the disease, reducing stress, successful experience in previous uses, improving physical condition, methods safety, family and friends' suggestions, and reducing the side effects of conventional treatments such as fatigue, nausea and vomiting, and diarrhea.

Among the 14 articles, five did not report information about the disclosure of CAM use with the physician. Disclosure to physicians among the remaining studies was 39 %.

### 3.4 Quality assessment of the included articles

The score range of the entered articles was between 4 and 13.5, and 10 out of 14 articles received a score of eight or more. The details of the scores are presented in Table 3.

**Table 3:** Details of scoring of included articles using quality assessment tool (QAT).

**Included studies**	**Study methods (4 points)**	**Sampling (2 points)**	**Reporting of participants’ characteristics (4 points)**	**CAM use (6 points)**	**Total score (16 points) (%)**
(Montazeri et al., 2005)	1	0	2	1	4 (25 %)
(Sajadian et al., 2005)	3	1	2.5	2	8.5 (53.1 %)
(Montazeri et al., 2007)	2	0	2.5	4	8.5 (53.1 %)
(Sajadian et al., 2009)	3	1	3	2	9 (56.2 %)
(Bordbar et al., 2016)	2	1	3	2	8 (50 %)
(Amirmoezi et al., 2017)	1	1	2.5	2	6.5 (40.6 %)
(Bazrafshani et al., 2019)	4	1	2.5	2	9.5 (59.3 %)
(Dehghan et al., 2019)	1	1	3	4	9 (56.2 %)
(Farahani et al., 2019)	2	1	1	2	6 (37.5 %)
(Jalili et al., 2020)	2	1	1.5	1	5.5 (34.3 %)
(Nejat et al., 2022)	2	1	3	2	8 (50 %)
(Dehghan et al., 2022)	2	2	3.5	6	13.5 (84.3 %)
(Dehghan et al., 2022)	2	2	3.5	6	13.5 (84.3 %)
(Asadi Sarvestani et al., 2022)	2	2	3.5	3	10.5 (65.6 %)

CAM: complementary and alternative medicine.

## 4 Discussion

In this study, we aimed to investigate the prevalence, techniques, and characteristics of CAM usage among cancer patients in Iran by reviewing relevant studies. A total of 14 articles were included, which were not covered by previous reviews. Notably, Iran ranked fourth in the number of studies after Germany, but no study from Iran was included in previous reviews (Horneber et al., 2012). Among the included studies, Kerman, Tehran, and Shiraz had the highest number of articles, whereas no studies were conducted in Isfahan, Mashhad, and Tabriz.

In comparing our study on CAM use among cancer patients in Iran with three previous studies, several noteworthy insights emerge. Firstly, our investigation revealed a relatively high prevalence of CAM use among Iranian cancer patients, with a weighted average of 51.83 %. This contrasts with the findings of the study by Markus Horneber et al., which reported a lower overall prevalence of 40 % across 18 countries (Horneber et al., 2011). The variation in CAM utilization rates could be attributed to cultural, regional, or healthcare system differences, highlighting the importance of context-specific considerations in understanding patient choices regarding integrative therapies.

Secondly, the diversity of CAM methods reported in our study, such as prayer, medicinal herbs, traditional treatments, bloodletting, and hydrotherapy, aligns with the global interest in diverse approaches to cancer care. Interestingly, the studies by Nicolas Calcagni et al. and Dana C. Mora et al. focused on specific subsets of CAM, namely manipulative and body-based practices, and CAM modalities to treat adverse effects in children and young adults, respectively (Calcagni et al., 2019, Mora et al., 2022). These studies underscore the need for nuanced investigations into particular categories of CAM, as they may yield distinct patterns and effectiveness in managing cancer-related symptoms.

The prevalence of CAM usage varies widely among different regions and cultures. For instance, Frass et al. (2012) reported that 32.2 % of the general population used CAM, ranging from 5 % in the United States to 74.8 % in South Korea. A study conducted in northern Iran (Babol) by Moeini et al. (2018) found that 71.46 % of the general population had used CAM at least once, which is similar to the prevalence reported in South Korea. In cancer patients, the prevalence of CAM usage ranges from 48 % to 51 %, and our study showed a prevalence of 51.83 % (Keene et al., 2019, Berretta et al., 2017).

Non-herbal supplements, massage, chiropractic, music therapy, and yoga were commonly used CAM methods reported in previous studies, whereas prayer, medicinal herbs, and Persian medicine treatments like bloodletting and cupping were more frequently used in Iran. The primary motivations for using CAM among cancer patients were to help cure cancer, reduce chemotherapy side effects, and improve physical and mental health (Bishop et al., 2010 Apr, Alsharif, 2021). Previous studies have identified female gender, younger age, higher education, and higher socioeconomic status as predisposing factors for CAM use, which was consistent with our study (Keene et al., 2019, Bishop et al., 2010 Apr). However, Montazeri's study reported higher age and lower education as predisposing factors for CAM use (Montazeri et al., 2005). Additional factors such as higher monthly income, living in urban areas, higher activity, and fear of cancer recurrence were also found to be predisposing factors for CAM use.

Only 39 % of the patients in our study disclosed their CAM usage to their physicians, which is lower than the rates reported in other studies (50–60 %) (Davis et al., 2012). Patients' fear of physicians' disapproval, lack of inquiry, or stigma associated with some CAM methods were identified as reasons for the low disclosure rates (Bello et al., 2012).

Various types of CAM are reportedly used by cancer patients worldwide (Cassileth and Deng, 2004). Mind-body techniques, such as meditation, yoga, and tai chi, are utilized to enhance mental and emotional well-being, reduce stress, and promote relaxation. These practices are considered to aid cancer patients in coping with the psychological and emotional effects of the disease, as well as alleviate symptoms such as pain, fatigue, and anxiety (Horneber et al., 2012). On the other hand, natural products encompass a variety of herbal supplements, vitamins, and other natural substances that are believed to have therapeutic effects. Among the most commonly used natural products among cancer patients are echinacea and ginseng. Manipulative and body-based practices constitute another type of CAM used by cancer patients, including chiropractic and massage therapy. Chiropractic therapy involves the manipulation of the spine and other joints to relieve pain and improve mobility, whereas massage therapy entails the manual manipulation of muscles and soft tissues to alleviate tension and enhance circulation. These practices are believed to be beneficial for cancer patients who experience pain, muscle tension, or stiffness resulting from their disease or treatment. In our study, praying was found to be the most prevalent type of CAM utilized by cancer patients, which can be classified as a mind–body intervention, followed by medicinal plants, similar to the commonly used types of CAM reported in other studies. However, practices such as bloodletting may not be as popular in European and American countries, where chiropractic and homeopathy may be more frequently used (Molassiotis et al., 2005).

### 4.1 Limitations

One limitation of this study was the lack of information on the exact duration and goals of CAM use in the included studies. Therefore, standard questionnaires with clear definitions of CAM use should be developed and utilized in future studies to enable meta-analysis and facilitate decision-making (Chijan et al., 2022). Additionally, the high heterogeneity among the studies made it impossible to perform a *meta*-analysis. Moreover, the studies included in this review had minor defects in quality evaluation, which may have affected the accuracy and reliability of the results. Therefore, caution is needed when interpreting the findings of this study. The limitation of sample size in our study causes a constraint on the generalizability of our findings. The wide range in reported CAM usage (4.6 %-100 %) reflects the heterogeneity in practices among different patient groups. Despite efforts to comprehensively review available literature, the relatively small sample size may limit the broader applicability of our conclusions to the entire spectrum of cancer patients in Iran.

### 4.2 Future research direction

Future research in the realm of complementary and alternative medicine (CAM) use among cancer patients in Iran should delve deeper into the factors influencing patient decisions, disclosure patterns to healthcare professionals, and the impact of cultural and regional variations on CAM preferences. Exploring the reasons behind the reported prevalence and specific modalities identified in our study could provide a more nuanced understanding of patient motivations and expectations. Additionally, investigating the efficacy and safety of prevalent CAM methods in the Iranian context is crucial for informing evidence-based guidelines and facilitating open communication between patients and healthcare providers. Longitudinal studies tracking changes in CAM utilization over time and in response to evolving healthcare practices would contribute to a dynamic understanding of patient preferences. Furthermore, collaborative research efforts between traditional and conventional healthcare practitioners can foster an integrative approach to cancer care in Iran, ensuring a comprehensive and culturally sensitive model that respects patients' beliefs and preferences while optimizing their overall well-being.

## 5 Conclusion

To our knowledge, this is the first systematic review of CAM use among cancer patients in Iran. The results of this study revealed that the most commonly used CAM methods among Iranian cancer patients were prayer, medicinal herbs, and Persian medicine treatments, which differs from the patterns seen in other studies. Furthermore, the rate of physician disclosure of CAM use was slightly lower than reported in other studies. In light of the potential risks and interactions of CAM methods with conventional cancer treatment, it is essential for physicians to acquire sufficient knowledge in this field and to provide appropriate guidance and counseling to their patients.

## Funding

This study was supported by a grant from Shiraz University of Medical Sciences (grant number: 26641).

## CRediT authorship contribution statement

**Mohammad Yusefi:** Data curation, Investigation, Software. **Hamid Reihani:** Data curation, Investigation, Writing – original draft, Visualization. **Mojtaba Heydari:** Data curation, Investigation, Methodology, Writing – review & editing. **Ramin Nasimi Doost Azgomi:** Data curation, Investigation, Writing – review & editing. **Mohammad Hashem Hashempur:** Data curation, Investigation, Software, Writing – original draft, Methodology, Supervision.

## Declaration of competing interest

The authors declare that they have no known competing financial interests or personal relationships that could have appeared to influence the work reported in this paper.

## Data Availability

No data was used for the research described in the article.
